# cAMP inhibits migration, ruffling and paxillin accumulation in focal adhesions of pancreatic ductal adenocarcinoma cells: Effects of PKA and EPAC^☆^

**DOI:** 10.1016/j.bbamcr.2013.06.011

**Published:** 2013-12

**Authors:** Alex Burdyga, Alan Conant, Lee Haynes, Jin Zhang, Kees Jalink, Robert Sutton, John Neoptolemos, Eithne Costello, Alexei Tepikin

**Affiliations:** a Department of Cellular and Molecular Physiology, The University of Liverpool, Crown Street, Liverpool L69 3BX, UK; b The Johns Hopkins University School of Medicine, Department of Neuroscience, 725 North Wolfe Street, Baltimore, MD 21205, USA; c The Netherlands Cancer Institute, Cell Biology I, Plesmanlaan 121, 1066 CX Amsterdam, The Netherlands; d NIHR Liverpool Pancreas Biomedical Research Unit, The University of Liverpool, Crown Street, Liverpool L69 3BX, UK; e Department of Molecular and Clinical Cancer Medicine, 6th Floor, Duncan Building, Daulby Street, Liverpool, L69 3GA, UK

**Keywords:** 6Bnz-cAMP, N6-benzoyl-cAMP, 8Br-cAMP, 8, 8-bromoadenosine-cAMP, 8pCPT, 8, 8-(4-chlorophenylthio)-2′-O-methyl-cAMP, AKAR4, A-kinase activity reporter four, DMEM, Dulbecco's modified Eagle's medium, EPAC, exchange protein activated by cAMP, FBS, foetal bovine serum, Frsk, forskolin, H84, CFP(nd)-EPAC(dDEP/CD)-Venus(d), IBMX, 3-isobutyl-1-methylxanthine, PBS, phosphate buffered solution, PDAC, pancreatic ductal adenocarcinoma, PKA, protein kinase A, cAMP, PKA, EPAC, Cell migration, Paxillin, Pancreatic ductal adenocarcinoma

## Abstract

We demonstrated that increasing intracellular cAMP concentrations result in the inhibition of migration of PANC-1 and other pancreatic ductal adenocarcinoma (PDAC) cell types. The rise of cAMP was accompanied by rapid and reversible cessation of ruffling, by inhibition of focal adhesion turnover and by prominent loss of paxillin from focal adhesions. All these phenomena develop rapidly suggesting that cAMP effectors have a direct influence on the cellular migratory apparatus. The role of two primary cAMP effectors, exchange protein activated by cAMP (EPAC) and protein kinase A (PKA), in cAMP-mediated inhibition of PDAC cell migration and migration-associated processes was investigated. Experiments with selective activators of EPAC and PKA demonstrated that the inhibitory effect of cAMP on migration, ruffling, focal adhesion dynamics and paxillin localisation is mediated by PKA, whilst EPAC potentiates migration.

## Highlights

• Increases in cAMP inhibit migration and invasion of pancreatic ductal adenocarcinoma cells.
• Increases in cAMP rapidly and reversibly inhibit ruffling.
• Increases in cAMP suppress the dynamics of focal adhesions and induce paxillin trafficking out of focal adhesions.
• The effects of cAMP on migration, ruffling and focal adhesions are mediated by PKA whilst EPAC potentiates migration.

## 1 Introduction

Pancreatic ductal adenocarcinoma (PDAC), a leading cause of cancer-related deaths world-wide, is characterised by widely disseminated metastatic disease at diagnosis. As a consequence most patients die within one year, and less than 5% of patients survive five years [37]. Global genomic analyses of individual pancreatic tumours and corresponding metastases have recently provided novel insights into the molecular characteristics of PDAC [4,42]. Moreover, it was recently demonstrated that early precursor cells, previously thought to lack invasive properties, appeared capable of invading and seeding distant sites [34,40]. Nonetheless, despite advances in our understanding of PDAC [8,9] the processes controlling metastasis formation have not been fully elucidated. Invasion and migration of cancer cells are among the processes essential for the formation of metastases [18,35]. In this study we aimed to investigate the effect of the ubiquitous second messenger cAMP on the migration of PDAC cells.

There are 3 primary cAMP sensors: PKA, EPAC and cAMP-activated ion channels [7]. Recent advances in the development of molecular probes based on fluorescence resonance energy transfer allow one to monitor intracellular cAMP concentration [33,41,43,44] and its downstream effects on EPAC activation [33,41] and PKA activity [12,13,45] in living cells. This is significant since changes in this signalling cascade can now be directly correlated with other cellular responses important for cell migration, e.g. actin dynamics at the leading edge of the cell and trafficking of proteins in and out of focal adhesions.

The effects of cAMP and its primary sensors on cancer cell migration are complex and cell-type specific: gradients of cAMP and PKA activity were shown to be necessary for migration of a number of cell types [21,25,29,32] (recently reviewed in [20]), however the inhibition of PKA strongly potentiated migration of mouse embryonic fibroblasts [6] and Clone A colon carcinoma cells [31]. Conversely, inhibition of PKA inhibited migration of MDA-MB-435 breast carcinoma cells [30]. Similar ambiguity exists for another primary cAMP effector: selective activation of EPAC inhibited the migration of PC-3 prostate carcinoma cells [16], MDCK-GFP-EPAC kidney epithelial cells [28] and H295 adrenocortical carcinoma cells [2], but potentiated migration of SK-Mel-2 melanoma cells [3]. These published studies indicate that the effects of cAMP and its effectors on migration/invasion can be very substantial and are variable, depending on the cell-type. The outcome of interfering with the cAMP signalling cascade is therefore difficult to predict and the effects need to be elucidated in each individual cell type.

This project was initiated by a serendipitous observation that increasing intracellular cAMP concentration strongly inhibited migration of PDAC cells. The study aimed to characterise this phenomenon and determine the primary cAMP effector(s) responsible for the observed inhibition; this is particularly relevant considering the reported high level expression of EPAC1 [27] and RIα subunit of PKA [15] in PDAC cells.

## 2 Materials and methods

### 2.1 Chemicals

Forskolin (Frsk) and 8-bromoadenosine (8Br-cAMP) were purchased from Tocris Biosciences, UK. 8-(4-Chlorophenylthio)-2′-O-methyl-cAMP (8pCPT), N6-benzoyl-cAMP (6Bnz-cAMP) and recently developed EPAC inhibitor (see [1]) 3-[5-(tert.-butyl)isoxazol-3-yl]-2-[2-(3-(chlorophenyl))hydrazono]-3-oxopropanenitrile (ESI-09) were acquired from BioLog, Germany. All other chemicals were from Sigma Aldrich, UK.

### 2.2 Cell culture

PDAC cell lines PANC-1, BxPC3, CAPAN-2, MiaPaca-2 (all obtained from the American Type Culture Collection) and SUIT-2 [23], were maintained in Dulbecco's modified Eagle's medium (DMEM), supplemented with 10% foetal bovine serum (FBS), penicillin (100 units/ml), streptomycin (100 μg/ml) and L-glutamine (0.29 mg/ml). All tissue culture reagents were purchased from Invitrogen (Paisley, UK). Cell cultures were maintained in standard humidified incubators (Wolf Laboratories) at 37 °C and 5% CO_2_. Prior to any imaging experiments, cells were washed with and transferred into Na^+^-HEPES-based extracellular solution (containing 140 mM NaCl, 4.7 mM KCl, 1.13 mM MgCl_2_, 10 mM HEPES (pH 7.4), 10 mM glucose, 1.8 mM CaCl_2_).

### 2.3 Plasmid constructs and transfection

Cells were transfected at approximately 60% confluency using PromoFectin reagent (PromoKine, UK), according to manufacturer's instructions.

EPAC based cAMP FRET sensor CFP(nd)-EPAC(dDEP/CD)-Venus(d) (H84; see http://research.nki.nl/jalinklab/Constructs.htm and [41]), was produced in the laboratory of Dr. K. Jalink (The Netherlands Cancer Institute). The effects of different concentrations of Frsk (adenylyl cyclase activator), IBMX (phosphodiesterase inhibitor) and membrane permeable cAMP analogues were tested in preliminary experiments on PANC-1 cells expressing H84. For example, we observed that 1 mM IBMX produced stronger H84 responses than 0.1 mM IBMX but further increase of IBMX concentration did not significantly increase the H84 response relative to 1 mM (Fig. S1). The concentrations of the individual compounds that produced clearly resolvable (but not saturating) responses of H84 were selected for further studies of the role of cAMP in PANC-1 cell migration (not shown). PKA sensor (AKAR4, containing PKA substrate sequence and Cerulean–Venus FRET pair) [12,13] was produced in the laboratory of Dr. J. Zhang (The Johns Hopkins University School of Medicine). PKA based cAMP FRET sensor (RII-L20-CFP + CAT-YFP) [26] was a gift from Dr. M. Zaccolo (University of Oxford). PKI-mCherry [39] was a gift from Dr. M. Ginsberg (University of California, San Diego). Paxillin–GFP was obtained from Addgene (Addgene plasmid 15233, PI Alan Horwitz [24]). Vinculin-Venus was from Addgene (Addgene plasmid 27300, Martin Schwartz, [17]). LifeAct-GFP was from Ibidi (Martinsried, Germany).

### 2.4 Confocal microscopy and FRET imaging

Leica AOBS TCS SP2 inverted laser scanning confocal microscope (LSCM) was used for cell imaging. Imaging experiments were conducted at 35–37 °C. Cells were grown to approximately 80% confluency in 35 mm glass-bottom dishes (MatTek, USA), and were placed into a custom made insert designed to accommodate the dish and allow changes of extracellular solution by perfusion.

Fluorescence of paxillin–GFP was excited using a 488 nm laser, and emission collected between 500 and 570 nm. In specific experiments aimed to reveal dynamics of focal adhesions (e.g. Fig. 3A and corresponding text in the Results section), all images were equally thresholded and subjected to single pixel size “remove outliers” function using ImageJ software to eliminate cytosolic component of fluorescence and highlight the position of focal adhesions. The dynamic turnover of the focal adhesions was then revealed by comparing two thresholded images that were recorded 1800 s apart: the images were merged together and structures with fluorescence above threshold on the first image were assigned the red colour, whilst the structures on the second image (that was taken 1800 s later) were coloured green. Therefore newly forming focal adhesions appear green on such merged images, whilst focal adhesions which have disassembled within the 1800 s period appear red. Some focal adhesions did not change within the 1800 s period and appear yellow as a result of the overlap between the red and green colours.

Paxillin–PSmOrange was from Addgene (Addgene plasmid 31923, PI Vladislav Verkhusha) [38]. Notably this plasmid encodes a photoswitchable version of mOrange; however in all our experiments, low intensity laser light was used to ensure that no photoswitching occurred and thus the probe was used as an ordinary mOrange tag with its typical excitation and emission spectra. PSmOrange was excited by a 543 nm laser and emission collected between 560 and 670 nm.

PKI-mCherry was excited using a 594 nm laser line, and emission collected between 605 and 690 nm.

Vinculin-Venus was excited using a 514 nm laser line and emission was collected between 530 nm and 590 nm.

LifeAct-GFP was excited using a 488 nm laser, and emission collected between 500 and 570 nm.

All FRET sensors utilised CFP (or Cerulean) and YFP (or Venus). In all experiments with FRET sensors a 405 nm laser line was used for excitation of fluorescence; fluorescence emission was collected for CFP or Cerulean between 450 and 490 nm, whilst for YFP or Venus it was between 520 and 590 nm. The ratio of fluorescence recorded using these wavelength bands was normalised to the baseline ratio and plotted against time to illustrate changes of cAMP levels or responses of its effectors. Quantification of recorded time-series images was performed using Leica Application Suite AF Lite software and ImageJ software.

The n numbers indicated in the Results section or figure legends are the number of individual cells in each experimental condition. Each type of experiment was repeated using at least 3 separate cell preparations.

### 2.5 Migration and invasion assays

Cell migration was assessed using 24-well Boyden chambers with 8 μM pore size (BD Biosciences, UK), and were used as specified by the manufacturer. DMEM supplemented with 1% FBS was placed into the top and bottom well of the Boyden chamber. Cells seeded into the top well were allowed to migrate through the porous membrane for 6 h in the incubator at 37 °C and 5% CO_2_. To test cell invasion, Matrigel covered Boyden chambers (BD Biosciences, UK), were used according to manufacturer specification. Cell migration and invasion were also assessed in conditions of asymmetric FBS distribution; in these experiments, bottom wells were filled with DMEM supplemented with 10% FBS, and the top wells with DMEM alone (i.e. no FBS added). Following 6 h of incubation, Boyden chambers were fixed using 100% methanol for 10 min at room temperature. Non-migrated cells were scraped away from the top surface of the Boyden chamber membrane. Boyden chambers were then placed into phosphate buffered solution (PBS), containing 137 mM NaCl, 2.68 mM KCl, 10.1 mM Na_2_HPO_4_, and 1.76 mM KH_2_PO_4_, and pH adjusted to 7.4 supplemented with 10 μg/ml RNase A for 30 min, which was followed by treatment with PBS solution supplemented with 100 μg/ml propidium iodide for 10 min at room temperature. Any remaining non-migrated cells were removed from the top surface of the membrane for a second time and the chambers were washed twice using PBS. The chambers were then placed into individual wells of 24-well plates containing 1 ml PBS per well and imaged using a 10 × objective and the Leica AOBS TCS SP2 confocal microscope. CellProfiler software [5] was used to identify and count the stained cells on the lower surface of the membrane. The n numbers indicated in the Results section or figure legends are the number of individual Boyden chambers for each experimental condition. Each type of experiment was repeated using at least 3 separate cell preparations. The results were analysed using Student's *t*-test, and p < 0.05 was considered statistically significant (and indicated by the symbol * on the figures). For each of the tested compounds the number of migrated cells was normalised to that in the control (untreated cells with appropriate vehicle) and presented as percentage of control. In data presentation the error margins represent standard error of mean.

### 2.6 Analysis of ruffling

A macro developed by Deming and colleagues [11] was used in conjunction with ImageJ software [36] to processes transmitted light series of images, reveal the ruffling and minimise background intensity fluctuation induced by the perfusion system. Processed image series were then used to produce kymographs from selected lines of interest, using the ImageJ plug-in ‘Multiple Kymograph’ developed by Jens Rietdorf and Arne Seitz (http://www.embl.de/eamnet/html/body_kymograph.html).

## 3 Results

### 3.1 Elevated cAMP inhibits PDAC cell migration and invasion

**Fig. 1:**
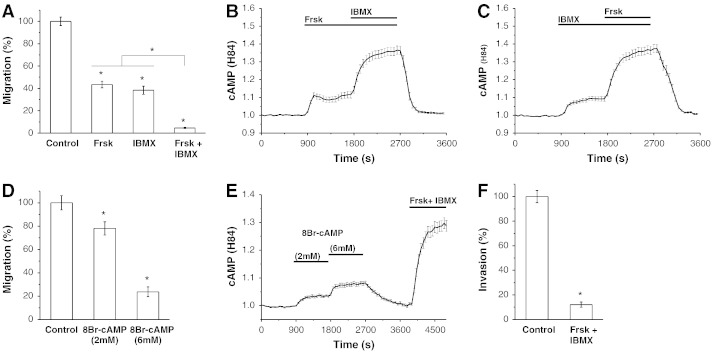
Increasing cAMP inhibits migration of PANC-1 cells. (A) PANC-1 cell migration, as measured by Boyden chamber assays (n = 18 for each condition), was significantly inhibited by 20 μM forskolin (Frsk), 1 mM IBMX and a combination of the two reagents. (B) The effect of 20 μM Frsk and a combination of 20 μM Frsk/1 mM IBMX on cytosolic cAMP levels in PANC-1 cells (n = 34). (C) The effect of 1 mM IBMX and a combination of 20 μM Frsk/1 mM IBMX on cytosolic cAMP levels in PANC-1 cells (n = 54). (D) PANC-1 cell migration, as measured by Boyden chamber assays (n = 9 for each condition), was significantly inhibited by 8Br-cAMP. (E) The effect of 8Br-cAMP on cytosolic cAMP levels in PANC-1 cells (n = 37); 20 μM Frsk and 1 mM IBMX were added at the end of experiments to saturate the probe. (F) PANC-1 cell invasion, as measured using Matrigel-coated Boyden chambers (n = 6 for each condition), was inhibited by 20 μM Frsk and 1 mM IBMX.

We observed that interventions increasing intracellular cAMP levels, such as treatment of cells with Frsk or IBMX, significantly inhibited PANC-1 cell migration as measured by Boyden chamber assays (Fig. 1A–C). Combined treatment with Frsk and IBMX produced stronger increases of cAMP (Fig. 1B and C) and stronger inhibition of migration (up to 95%) than the individual substances alone (Fig. 1A). Increasing intracellular cAMP with Frsk and IBMX also strongly inhibited the migration of SUIT-2, CAPAN-2, BxPC3 and MiaPaca-2 cell lines (Fig. S2).

The membrane-permeable cAMP analogue, 8Br-cAMP- also inhibited PANC-1 cell migration, confirming that the observed inhibition of migration is likely to be cAMP-mediated (Fig. 1D). The strength of the inhibition correlated with the response of the cAMP sensor (Fig. 1A-E). Interestingly, high concentrations of 8Br-cAMP were necessary to produce the inhibition of migration and to induce significant fluorescence changes in the cAMP sensor (see Fig. 1E). This could be due to the relatively low permeability of this analogue (see http://www.biolog.de/technical-info/lipophilicity-data/). Another contributing factor could be the high activity of phosphodiesterases in this cell type.

Increasing intracellular cAMP with Frsk and IBMX strongly suppressed PANC-1 cell invasion (Fig. 1F). Migration and invasion in conditions of asymmetrical FBS distribution were also strongly inhibited (Fig. S3). The results of these experiments are consistent with the reports of cAMP-mediated inhibition of migration in other cancer cell types (e.g. [2,7]), but note that some studies (e.g. [30]) report duality in the actions of cAMP i.e. cAMP can be required for migration or inhibit migration (depending on the mechanism of its generation; recently reviewed in [20]).

### 3.2 The effect of cAMP on ruffles

IBMX resulted in a decrease of actin density at the leading edge of the cell and cessation of ruffling (  LifeAct-GFP). The inhibition of ruffling by Frsk and IBMX was also recorded in more than 50 untransfected PANC-1 cells (not shown). Simultaneous measurements of cAMP and visualisation of ruffles using a kymograph approach showed that ruffling is very effectively and rapidly inhibited following cAMP increase (importantly  

### 3.3 The effect of cAMP on focal adhesion turnover and paxillin localisation

Turnover of focal adhesions is important for migration [22]. In our experiments focal adhesions were revealed using fluorescently-labelled paxillin (Fig. 3). In migratory cells that continuously formed new focal adhesions before application of Frsk and IBMX, treatment with Frsk and IBMX stopped the formation of new focal adhesions (Fig. 3A, n = 5). In these experiments we observed apparent loss of focal adhesions and/or significant decrease of paxillin content of focal adhesions upon Frsk and IBMX treatment (Fig. 3A). Removal of Frsk and IBMX restored turnover of focal adhesions and increased concentration of paxillin–GFP in focal adhesions (Fig. 3A).

    

Inhibition of the turnover of focal adhesions was also revealed in PANC-1 cells expressing fluorescently-labelled vinculin (Fig. S5).

The rapidity of these responses suggested that the cAMP-induced inhibition of migration and invasion of PDAC cells are likely to be mediated by direct action of cAMP effector(s) on the migratory apparatus of the cells (rather than via cAMP-dependent changes in gene expression). PKA and EPAC are two prominent cAMP effectors. Therefore we next tested their roles in cAMP-induced inhibition of cell migration.

### 3.4 The effects of cAMP on ruffles, focal adhesions and cell migration are likely to be mediated by PKA

As expected, PKA activity (measured with AKAR4) increased rapidly following Frsk and IBMX addition (Fig. 4A, n = 9). Expression of PKI-mCherry is, to our knowledge, the most specific way of inhibiting PKA activity; in our experiments the expression of PKI-mCherry completely inhibited PKA activation by Frsk and by a combination of Frsk and IBMX (Fig. 4B). PKI-mCherry expression also effectively prevented Frsk and IBMX-induced cessation of ruffling (Fig. 4C) and partially prevented paxillin trafficking from focal adhesions (Fig. 4D). Importantly, expression of PKI-mCherry significantly (albeit incompletely) blocked the inhibition of migration in response to Frsk and IBMX (Fig. 4E). It should also be noted that expression of PKI-mCherry alone has a tendency to potentiate migration of PANC-1 cells but in this case the effect was not statistically significant (n = 6).

The effects of PKI-mCherry expression suggest that cAMP actions on ruffling, paxillin trafficking and migration are likely to be mediated by PKA. It was, however, important to test the role of another cAMP effector, EPAC.

The selective activator of EPAC 8pCPT [14] applied at 300 μM had a strong (albeit not saturating) effect on the EPAC-based FRET sensor (Fig. 5A) and had no resolvable effect on PKA activity (Fig. 5B). Importantly this selective EPAC activator moderately potentiated rather than inhibited PANC-1 cell migration (Fig. 5C) and had no resolvable effect on ruffling or paxillin trafficking (n = 10 and n = 11 correspondingly). Consistent with this result the inhibition of EPAC with recently developed inhibitor ESI-09, suppressed migration of PANC-1 cells (Fig. S8A, n = 6). Furthermore, the application of ESI-09 did not reduce the inhibition of migration by Frsk and IBMX (Fig. S8B, n = 6); this clearly contrasts with the effect of PKA inhibitor.

The novel finding (acceleration of migration by EPAC activator) observed in our experiments in PDAC cells is consistent with the reported potentiating action of EPAC on the migration/invasion of a melanoma cell line [3]. Importantly, the observed potentiation of migration supports the conclusion, based on experiments with a recently identified inhibitor of EPAC [1], that EPAC (and specifically EPAC1) is important for migration of PDAC cells and that EPAC inhibition suppresses migration.

On the contrary, the selective PKA activator N6-benzoyl-cAMP (6Bnz-cAMP) induced PKA responses (Fig. 5D and Fig. S6), had no resolvable effect on the EPAC-based sensor (Fig. 5E) and suppressed cell migration (Fig. 5F). These experiments indicate that EPAC and PKA have opposing effects on the migration of PANC-1 cells and that the inhibitory effect of cAMP on PANC-1 cell migration is likely to be mediated by PKA.

## 4 Discussion

Understanding the mechanisms regulating migration of PDAC cells is important for elucidating the processes leading to metastasis formation in this lethal form of cancer. We investigated the role of cAMP signalling in the regulation of migration and observed that increasing cAMP concentration resulted in the inhibition of migration of all tested PDAC cell lines. Previous studies have shown that the effect of cAMP on migration of cancer cells is ambivalent — migration of some cell types is inhibited by cAMP increase whilst other cell types require cAMP signalling for efficient migration (reviewed in [19,20]). In this study we have tested a number of PDAC cells lines which have diverse genetic background and origin [10], however, all these PDAC cell lines displayed inhibition of migration as a result of cAMP elevation, suggesting that this phenomenon is common for different populations of cancer cells derived from this type of cancer. The observation that an increase of cAMP strongly inhibits migration of PANC-1 and other PDAC cell types suggests that this phenomenon could be exploited to develop treatments capable of delaying the progression of PDAC.

An important element of this study is that we extensively utilised an EPAC-based cAMP sensor (H84) [41] and a PKA sensor (AKAR4) [12,13] to correlate the cAMP levels and the status of its effectors with the observed changes in migration, ruffling, focal adhesions and paxillin trafficking. These measurements revealed that different manoeuvres, which increase cAMP (inhibition of phosphodiesterases, stimulation of adenylate cyclases or application of membrane permeant cAMP analogues) all resulted in the inhibition of migration and that the inhibition of migration depended on the amplitude of cAMP increase. The simultaneous measurements of cAMP levels and visualisation of membrane ruffling highlighted synchronicity of the cAMP elevation and the inhibition of ruffling, and indicated that the inhibition develops well before the saturation of the cAMP sensor. We also observed remarkable synchronicity of cAMP rise and paxillin trafficking out of focal adhesions. Such observations certainly reaffirm the causative relationships between the cAMP increase and modification of cellular migratory apparatus. Measurements utilising H84 and AKAR4 provided strong evidence that the cAMP analogue, expected to be EPAC selective, indeed activated EPAC and did not activate PKA and vice versa for PKA selective cAMP analogue. Finally, remarkably efficient inhibition of PKA responses by expressed PKI was confirmed by experiments with cells expressing AKAR4. It should be noted, however, that whilst PKI effectively inhibits global PKA responses (reported by AKAR4) some form of localised PKA signalling could still be maintained and contribute to the partial inhibition of migration in Frsk and IBMX treated cells expressing PKI.

The findings of our study show that the cAMP-induced inhibition of migration of PDAC cells is mediated by PKA and that the activation of EPAC has an opposing action.

These observations suggest that the development/optimisation of a treatment protocol involving simultaneous inhibition of EPAC and activation of PKA could be necessary to effectively inhibit PDAC cell migration in vivo and ultimately suppress the formation of metastases.

The following are the supplementary data related to this article.

Supplementary material.

**Fig. 2:**
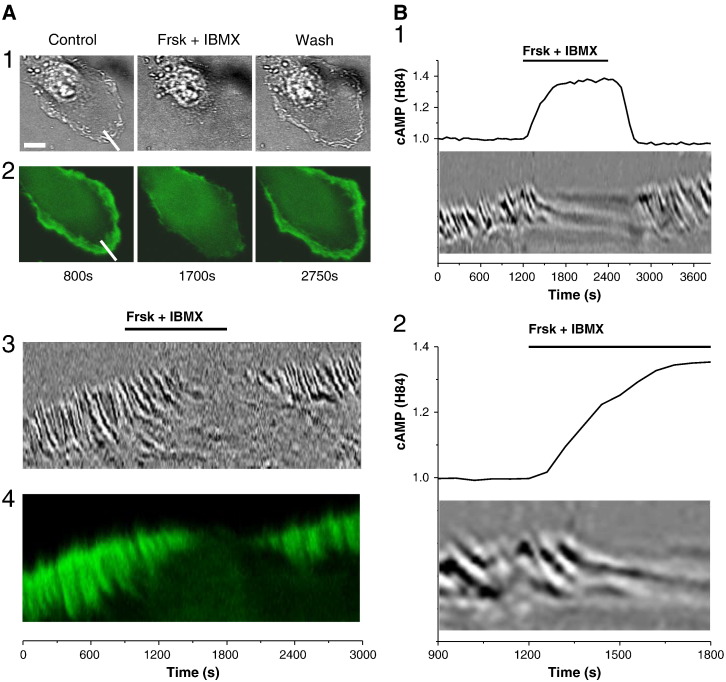
Treatment with forskolin and IBMX inhibits ruffle formation in PANC-1 cells. (A) Effect of 20 μM forskolin (Frsk) and 1 mM IBMX on ruffling and actin dynamics in lamellipodium of a PANC-1 cell. (A1) Transmitted light images of the cell before, during and after the treatment with Frsk and IBMX. The changes of ruffling and actin dynamics shown in (A3) and (A4) were measured along the line drawn across the plasma membrane region (shown in A1 and A2). Scale bar represents 10 μM. (A2) The fluorescence images of LifeAct-GFP expressed in the same cell. (A3) The ruffle formation plot (a kymograph) shows transmitted light intensity measured along the line drawn across the plasma membrane region (shown in A1) and plotted against time. Note the disappearance of ruffles following treatment with Frsk and IBMX. Removal of Frsk and IBMX from the extracellular solution restored the ruffling. (A4) The fluorescence of LifeAct-GFP measured in the same cell along the line drawn across the plasma membrane region (shown in A2; the location of the line is the same as in A1) and plotted against time. (B) Correlation between cAMP levels and ruffle formation. (B1) The kymograph shown in the lower part of the figure was constructed using the same procedure as illustrated in part A of this figure. Ruffle dynamics were monitored simultaneously with cAMP measurements (using H84) and displayed against the same time points as cAMP measurements (n = 3). (B2) Shows a fragment of recordings displayed in B1 on expanded time scale. Note the correlation between the cAMP increase induced by 20 μM Frsk and 1 mM IBMX and the cessation of ruffling.

**Fig. 3:**
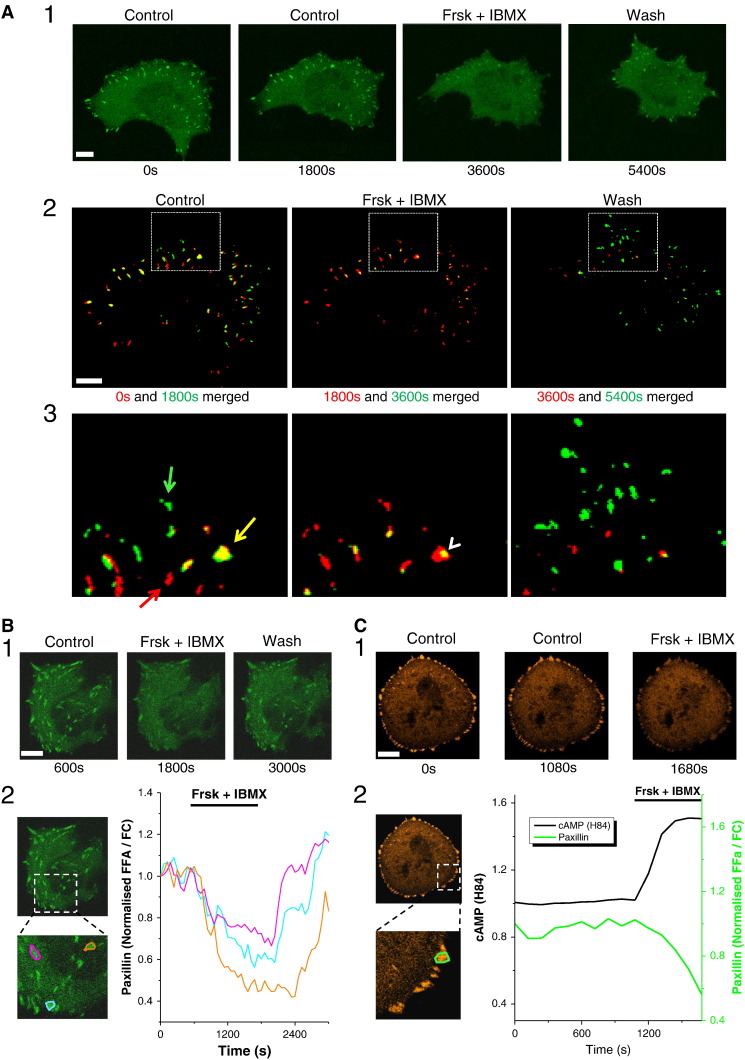
Increasing cAMP induces paxillin trafficking out of focal adhesions and inhibits turnover of focal adhesions. (A) Inhibition of focal adhesion turnover and loss of paxillin from focal adhesions in migrating PANC-1 cells. (A1) Cells were transfected with paxillin–GFP and (after 1800 s control period) subjected to 20 μM forskolin (Frsk) + 1 mM IBMX (from 1800 s to 3600 s), this was followed by a wash period (from 3600 s to 5400 s). Note the decrease of the number and fluorescence intensity of paxillin–GFP-labelled structures during Frsk + IBMX treatment. Scale bar represents 10 μm. (A2) The four images from A1 were thresholded using ImageJ (see Materials and methods) in order to reveal focal adhesions. The two thresholded images from the control period (0 s and 1800 s, see left panel) were assigned red and green colours respectively and merged together using ImageJ; thus newly formed focal adhesions appear green, whilst focal adhesions which have disassembled within the 1800 s period appear red. Some focal adhesions (or parts of focal adhesions) did not change within the 1800 s period and appear yellow as a result of the overlap between the red and green colours. The same process was used to merge thresholded images from 1800 s to 3600 s during the period of Frsk + IBMX application (see central panel). 1800 s image was assigned red colour this time, whilst 3600 s image was coded green. The same process was used again to merge images recorded at 3600 s and 5400 s (i.e. taken during the wash period; see right panel). Scale bar represents 10 μm. (A3) Shows expanded fragment from A2 (outlined by a box on A2). On the left panel green arrow shows an example of newly formed paxillin–GFP-labelled focal adhesion; red arrow shows an example of focal adhesion that disappeared during the first 1800 s of the experiment; yellow arrow shows an example of focal adhesion that remained largely unchanged. Note the disappearance of paxillin–GFP-labelled focal adhesions during Frsk + IBMX application (central panel; focal adhesion that disappeared during the period of the application is shown by red colour, an example of focal adhesion that was reduced in size but still present at the end of the treatment is shown by white arrowhead). Finally, removal of Frsk + IBMX restored the formation of new focal adhesions (green-coloured structures on the right panel). (B) Images of a stationary cell transfected with paxillin–GFP and treated with 20 μM Frsk + 1 mM IBMX. (B1) Shows reversible decrease of fluorescence in focal adhesions upon addition of Frsk + IBMX (see also Movie S2). Scale bar corresponds to 10 μm. (B2) Top left panel shows the same cell as in B1, with the outlined region shown as an expanded fragment on the lower left panel. Lower left panel shows the fragment with the highlighted regions of interest (containing focal adhesions) selected for analysis. The fluorescence recorded from individual regions of interests each containing a single focal adhesion (F_Fa_, shown in the left panel) was divided by fluorescence recorded from regions of the cell devoid of focal adhesions (F_C_) and plotted against time. Colours of the traces in the right panel correspond to the colours of the outlines of the regions of interest shown in the left panel. (C) Correlation between cAMP levels and paxillin trafficking in PANC-1 cells. (C1) Images of the cell transfected with paxillin–PSmOrange. Note the decrease of fluorescence in focal adhesions upon addition of 20 μM forskolin (Frsk) and 1 mM IBMX. Scale bar corresponds to 10 μm. (C2) Top left panel shows the same cell as in C1, with the outlined region shown as an expanded fragment on the lower left panel. Lower left panel shows the fragment with the highlighted region of interest (containing single focal adhesion) selected for analysis. The fluorescence recorded from the region containing the focal adhesion (F_Fa_) was divided by fluorescence recorded from the region of the cell devoid of focal adhesions (F_C_) and plotted against time (green line). The cAMP levels (H84 response) were measured from the entire cell and displayed against the time on the same graph (black line).

**Fig. 4:**
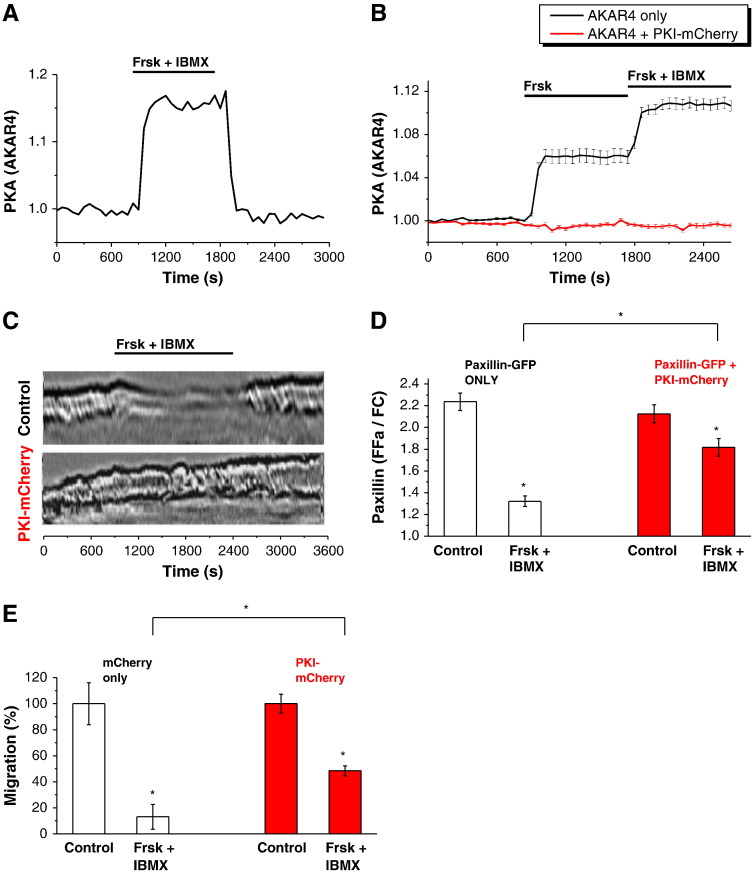
Modulating PKA activity affects migration, ruffling and paxillin trafficking in PANC-1 cells. (A) Shows PKA activation measured using AKAR4. The PKA activation was induced by 20 μM forskolin (Frsk) and 1 mM IBMX. (B) Expression of PKI-mCherry inhibits PKA activation. The black trace shows the response of AKAR4 (i.e. changes of PKA activity) induced by 10 μM Frsk and the combination of 20 μM Frsk and 1 mM IBMX in control cells (i.e. cells that are not transfected with PKI-mCherry, n = 46). The red trace (displaying AKAR4 FRET in cells transfected with PKI-mCherry) showed that PKA activation, either by 10 μM Frsk or (crucially) by the combination of 20 μM Frsk and 1 mM IBMX, is blocked (n = 51). (C) Expression of PKI-mCherry prevents inhibition of ruffling induced by 20 μM Frsk and 1 mM IBMX. The figure contains kymographs illustrating ruffling of a cell transfected with PKI-mCherry (lower panel; the majority of transfected cells (30 out 36) continued to ruffle in the presence of Frsk and IBMX) and of an untransfected control cell (upper panel; ruffling inhibited upon application of Frsk and IBMX, n > 50). (D) Expression of PKI-mCherry partially inhibits the trafficking of paxillin out of focal adhesions induced by 20 μM Frsk and 1 mM IBMX. The bars in the graph show the fluorescence recorded from regions of interests containing focal adhesions (F_Fa_) divided by fluorescence recorded from the region of the cell devoid of focal adhesions (F_C_). Frsk + IBMX represent measurements taken after 30 min of treatment with these compounds. The bars on the left represent measurements taken from cells expressing paxillin–GFP (n = 23); the bars on the right (shown in red) represent cells expressing paxillin–GFP and PKI-mCherry (n = 29). (E) Expression of PKI-mCherry partially prevents the inhibition of migration induced by 20 μM Frsk and 1 mM IBMX. Migration was measured by Boyden chamber assays. The bars on the left illustrate migration of cells expressing mCherry, whilst the bars on the right (shown in red) represent cells expressing PKI-mCherry (n = 6 for all conditions, the protective effect of PKI-mCherry expression was statistically significant).

**Fig. 5:**
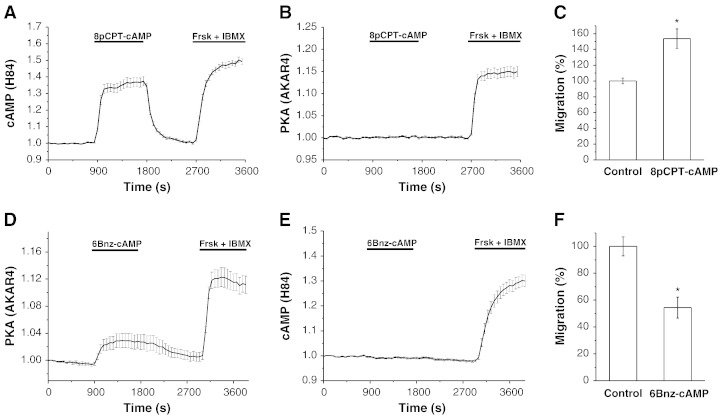
Effects of selective activators of EPAC and PKA on the migration of PANC-1 cells. (A) Response of the EPAC-based FRET sensor H84 expressed in PANC-1 cells to 300 μM 8-(4-chlorophenylthio)-2′-O-methyl-cAMP (8pCPT, n = 30). Here and in (B), (D), and (E) 20 μM forskolin (Frsk) and 1 mM IBMX were applied at the end of the experiments to saturate the FRET probes. (B) FRET of AKAR4 (PKA activity reporter) did not change upon the application of 300 μM 8pCPT (n = 21). (C) Migration, measured using Boyden chamber assays, was significantly potentiated by 300 μM 8pCPT (n = 16 for both control and 8pCPT treated cells). Note that migration was not significantly modified by lower concentrations (10 μM and 30 μM) of 8pCPT (see Fig. S7). (D) Response of AKAR4 to the application of 6 mM N6-benzoyl-cAMP (6Bnz-cAMP, n = 28). (E) FRET of EPAC-based sensor H84 did not change upon the application of 6 mM 6Bnz-cAMP (n = 29). (F) Migration of PANC-1 cells measured using Boyden chamber assays was significantly inhibited by 6 mM 6Bnz-cAMP (n = 7).

Movie S1 Treatment with forskolin and IBMX inhibits ruffle formation and actin dynamics in PANC-1 cells.
Effect of 20 μM forskolin (Frsk) and 1 mM IBMX on ruffling and actin dynamics of a PANC-1 cell. This movie accompanies Fig. 2A of the main part of the manuscript. Top left part (1) shows transmitted light movie of ruffle formation in the cell before, during and after treatment with Frsk and IBMX. Scale bar represents 10 μm. The kymograph illustrating the ruffling (shown in the lower left part of the same section) was recorded along the line drawn across the plasma membrane region depicted in the movie. The right part (2) shows the movie (recorded in fluorescence light) illustrating the dynamics of LifeAct-GFP expressed in the same cell. Lower part of this section shows the fluorescence of LifeAct-GFP measured in the same cell along the line drawn across the plasma membrane region (depicted in the movie) and plotted against time. Note the disappearance of ruffles (1) and cessation of actin dynamics (2) following treatment with forskolin and IBMX. Removal of forskolin and IBMX from the extracellular solution restored the ruffling and actin dynamics.

Movie S2 Forskolin and IBMX induce paxillin trafficking from focal adhesions.
Effect of 20 μM forskolin (Frsk) and 1 mM IBMX on the distribution of paxillin–GFP fluorescence. This movie accompanies Fig. 2C of the main part of the manuscript. Note the decrease of fluorescence in focal adhesions following the application of Frsk and IBMX and increase of the fluorescence after washing off of these compounds. Scale bar corresponds to 10 μm.

## Sources of funding

A.B. is an Medical Research Council (UK)-funded PhD student; the study was also supported by the National Institute for Health Research (UK) grant to the NIHR Liverpool Pancreas Biomedical Research Unit.

## Conflict of interest

None declared.

## References

[1] Almahariq M., Tsalkova T., Mei F.C., Chen H., Zhou J., Sastry S.K., Schwede F., Cheng X. (2013). A novel EPAC-specific inhibitor suppresses pancreatic cancer cell migration and invasion. Mol. Pharmacol..

[2] Aumo L., Rusten M., Mellgren G., Bakke M., Lewis A.E. (2010). Functional roles of protein kinase A (PKA) and exchange protein directly activated by 3′,5′-cyclic adenosine 5′-monophosphate (cAMP) 2 (EPAC2) in cAMP-mediated actions in adrenocortical cells. Endocrinology.

[3] Baljinnyam E., De Lorenzo M.S., Xie L.H., Iwatsubo M., Chen S., Goydos J.S., Nowycky M.C., Iwatsubo K. (2010). Exchange protein directly activated by cyclic AMP increases melanoma cell migration by a Ca2+-dependent mechanism. Cancer Res..

[4] Campbell P.J., Yachida S., Mudie L.J., Stephens P.J., Pleasance E.D., Stebbings L.A., Morsberger L.A., Latimer C., McLaren S., Lin M.L., McBride D.J., Varela I., Nik-Zainal S.A., Leroy C., Jia M., Menzies A., Butler A.P., Teague J.W., Griffin C.A., Burton J., Swerdlow H., Quail M.A., Stratton M.R., Iacobuzio-Donahue C., Futreal P.A. (2010). The patterns and dynamics of genomic instability in metastatic pancreatic cancer. Nature.

[5] Carpenter A.E., Jones T.R., Lamprecht M.R., Clarke C., Kang I.H., Friman O., Guertin D.A., Chang J.H., Lindquist R.A., Moffat J., Golland P., Sabatini D.M. (2006). Cell profiler: image analysis software for identifying and quantifying cell phenotypes. Genome Biol..

[6] Chen L., Zhang J.J., Huang X.Y. (2008). cAMP inhibits cell migration by interfering with Rac-induced lamellipodium formation. J. Biol. Chem..

[7] Cheng X., Ji Z., Tsalkova T., Mei F. (2008). Epac and PKA: a tale of two intracellular cAMP receptors. Acta Biochim. Biophys. Sin. (Shanghai).

[8] Costello E., Greenhalf W., Neoptolemos J.P. (2012). New biomarkers and targets in pancreatic cancer and their application to treatment. Nat. Rev. Gastroenterol. Hepatol..

[9] Costello E., Neoptolemos J.P. (2011). Pancreatic cancer in 2010: new insights for early intervention and detection. Nat. Rev. Gastroenterol. Hepatol..

[10] Deer E.L., Gonzalez-Hernandez J., Coursen J.D., Shea J.E., Ngatia J., Scaife C.L., Firpo M.A., Mulvihill S.J. (2010). Phenotype and genotype of pancreatic cancer cell lines. Pancreas.

[11] Deming P.B., Campbell S.L., Baldor L.C., Howe A.K. (2008). Protein kinase A regulates 3-phosphatidylinositide dynamics during platelet-derived growth factor-induced membrane ruffling and chemotaxis. J. Biol. Chem..

[12] Depry C., Allen M.D., Zhang J. (2011). Visualization of PKA activity in plasma membrane microdomains. Mol. Biosyst..

[13] Depry C., Zhang J. (2010). Visualization of kinase activity with FRET-based activity biosensors. Curr. Protoc. Mol. Biol..

[14] Enserink J.M., Christensen A.E., de Rooij J., van Triest M., Schwede F., Genieser H.G., Doskeland S.O., Blank J.L., Bos J.L. (2002). A novel Epac-specific cAMP analogue demonstrates independent regulation of Rap1 and ERK. Nat. Cell Biol..

[15] Farrow B., Rychahou P., Murillo C., O'Connor K.L., Iwamura T., Evers B.M. (2003). Inhibition of pancreatic cancer cell growth and induction of apoptosis with novel therapies directed against protein kinase A. Surgery.

[16] Grandoch M., Rose A., ter Braak M., Jendrossek V., Rubben H., Fischer J.W., Schmidt M., Weber A.A. (2009). Epac inhibits migration and proliferation of human prostate carcinoma cells. Br. J. Cancer.

[17] Grashoff C., Hoffman B.D., Brenner M.D., Zhou R., Parsons M., Yang M.T., McLean M.A., Sligar S.G., Chen C.S., Ha T., Schwartz M.A. (2010). Measuring mechanical tension across vinculin reveals regulation of focal adhesion dynamics. Nature.

[18] Hanahan D., Weinberg R.A. (2011). Hallmarks of cancer: the next generation. Cell.

[19] Howe A.K. (2004). Regulation of actin-based cell migration by cAMP/PKA. Biochim. Biophys. Acta.

[20] Howe A.K. (2011). Cross-talk between calcium and protein kinase A in the regulation of cell migration. Curr. Opin. Cell Biol..

[21] Howe A.K., Baldor L.C., Hogan B.P. (2005). Spatial regulation of the cAMP-dependent protein kinase during chemotactic cell migration. Proc. Natl. Acad. Sci. U. S. A..

[22] Huttenlocher A., Horwitz A.R. (2011). Integrins in cell migration. Cold Spring Harb. Perspect. Biol..

[23] Iwamura T., Katsuki T., Ide K. (1987). Establishment and characterization of a human pancreatic cancer cell line (SUIT-2) producing carcinoembryonic antigen and carbohydrate antigen 19-9. Jpn. J. Cancer Res..

[24] Laukaitis C.M., Webb D.J., Donais K., Horwitz A.F. (2001). Differential dynamics of alpha 5 integrin, paxillin, and alpha-actinin during formation and disassembly of adhesions in migrating cells. J. Cell Biol..

[25] Lim C.J., Kain K.H., Tkachenko E., Goldfinger L.E., Gutierrez E., Allen M.D., Groisman A., Zhang J., Ginsberg M.H. (2008). Integrin-mediated protein kinase A activation at the leading edge of migrating cells. Mol. Biol. Cell.

[26] Lissandron V., Terrin A., Collini M., D'alfonso L., Chirico G., Pantano S., Zaccolo M. (2005). Improvement of a FRET-based indicator for cAMP by linker design and stabilization of donor–acceptor interaction. J. Mol. Biol..

[27] Lorenz R., Aleksic T., Wagner M., Adler G., Weber C.K. (2008). The cAMP/Epac1/Rap1 pathway in pancreatic carcinoma. Pancreas.

[28] Lyle K.S., Raaijmakers J.H., Bruinsma W., Bos J.L., de Rooij J. (2008). cAMP-induced Epac–Rap activation inhibits epithelial cell migration by modulating focal adhesion and leading edge dynamics. Cell. Signal..

[29] McKenzie A.J., Campbell S.L., Howe A.K. (2011). Protein kinase A activity and anchoring are required for ovarian cancer cell migration and invasion. PLoS One.

[30] O'Connor K.L., Mercurio A.M. (2001). Protein kinase A regulates Rac and is required for the growth factor-stimulated migration of carcinoma cells. J. Biol. Chem..

[31] O'Connor K.L., Nguyen B.K., Mercurio A.M. (2000). RhoA function in lamellae formation and migration is regulated by the alpha6beta4 integrin and cAMP metabolism. J. Cell Biol..

[32] Paulucci-Holthauzen A.A., Vergara L.A., Bellot L.J., Canton D., Scott J.D., O'Connor K.L. (2009). Spatial distribution of protein kinase A activity during cell migration is mediated by A-kinase anchoring protein AKAP Lbc. J. Biol. Chem..

[33] Ponsioen B., Zhao J., Riedl J., Zwartkruis F., van der Krogt G., Zaccolo M., Moolenaar W.H., Bos J.L., Jalink K. (2004). Detecting cAMP-induced Epac activation by fluorescence resonance energy transfer: Epac as a novel cAMP indicator. EMBO Rep..

[34] Rhim A.D., Mirek E.T., Aiello N.M., Maitra A., Bailey J.M., McAllister F., Reichert M., Beatty G.L., Rustgi A.K., Vonderheide R.H., Leach S.D., Stanger B.Z. (2012). EMT and dissemination precede pancreatic tumor formation. Cell.

[35] Ridley A.J. (2011). Life at the leading edge. Cell.

[36] Schneider C.A., Rasband W.S., Eliceiri K.W. (2012). NIH Image to ImageJ: 25 years of image analysis. Nat. Methods.

[37] Siegel R., Naishadham D., Jemal A. (2013). Cancer statistics, 2013. CA Cancer J. Clin..

[38] Subach O.M., Entenberg D., Condeelis J.S., Verkhusha V.V. (2012). A FRET-facilitated photoswitching using an orange fluorescent protein with the fast photoconversion kinetics. J. Am. Chem. Soc..

[39] Tkachenko E., Sabouri-Ghomi M., Pertz O., Kim C., Gutierrez E., Machacek M., Groisman A., Danuser G., Ginsberg M.H. (2011). Protein kinase A governs a RhoA–RhoGDI protrusion–retraction pacemaker in migrating cells. Nat. Cell Biol..

[40] Tuveson D.A., Neoptolemos J.P. (2012). Understanding metastasis in pancreatic cancer: a call for new clinical approaches. Cell.

[41] van der Krogt G.N., Ogink J., Ponsioen B., Jalink K. (2008). A comparison of donor–acceptor pairs for genetically encoded FRET sensors: application to the Epac cAMP sensor as an example. PLoS One.

[42] Yachida S., Jones S., Bozic I., Antal T., Leary R., Fu B., Kamiyama M., Hruban R.H., Eshleman J.R., Nowak M.A., Velculescu V.E., Kinzler K.W., Vogelstein B., Iacobuzio-Donahue C.A. (2010). Distant metastasis occurs late during the genetic evolution of pancreatic cancer. Nature.

[43] Zaccolo M., De G.F., Cho C.Y., Feng L., Knapp T., Negulescu P.A., Taylor S.S., Tsien R.Y., Pozzan T. (2000). A genetically encoded, fluorescent indicator for cyclic AMP in living cells. Nat. Cell Biol..

[44] Zaccolo M., Pozzan T. (2002). Discrete microdomains with high concentration of cAMP in stimulated rat neonatal cardiac myocytes. Science.

[45] Zhang J., Ma Y., Taylor S.S., Tsien R.Y. (2001). Genetically encoded reporters of protein kinase A activity reveal impact of substrate tethering. Proc. Natl. Acad. Sci. U. S. A..

