# Supplementary material for: cAMP inhibits migration, ruffling and paxillin accumulation in focal adhesions of pancreatic ductal adenocarcinoma cells: Effects of PKA and EPAC
Source: Biochim Biophys Acta. 2013 Dec;1833(12):2664–72. doi: 10.1016/j.bbamcr.2013.06.011 (PMC3898478; doi:10.1016/j.bbamcr.2013.06.011)
Supplement: Supplementary file 1 — Supplementary material. [file mmc1.pdf]

SUPPLEMENTARY MATERIAL

cAMP inhibits migration, ruffling and paxillin accumulation in focal adhesions of pancreatic ductal adenocarcinoma cells: Effects of PKA and EPAC

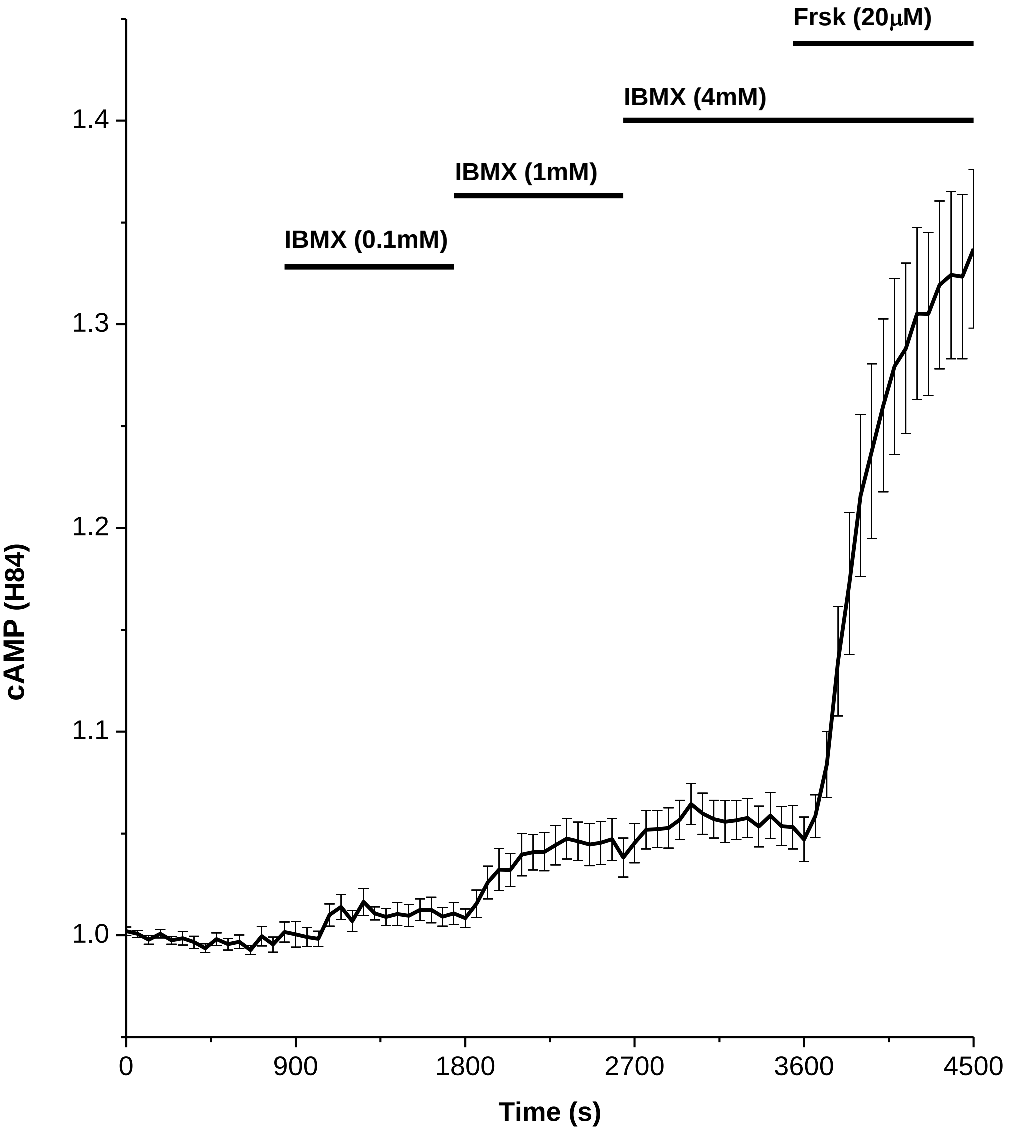

Fig. S1. Response of EPAC-based FRET sensor to different concentrations of IBMX.
Response of the EPAC-based FRET sensor H84 [1] expressed in PANC-1 cells to 0.1mM, 1mM and 4mM of IBMX (n= 8). 20µM forskolin (Frsk) and 4mM IBMX were added at the end of experiments to saturate the probe.

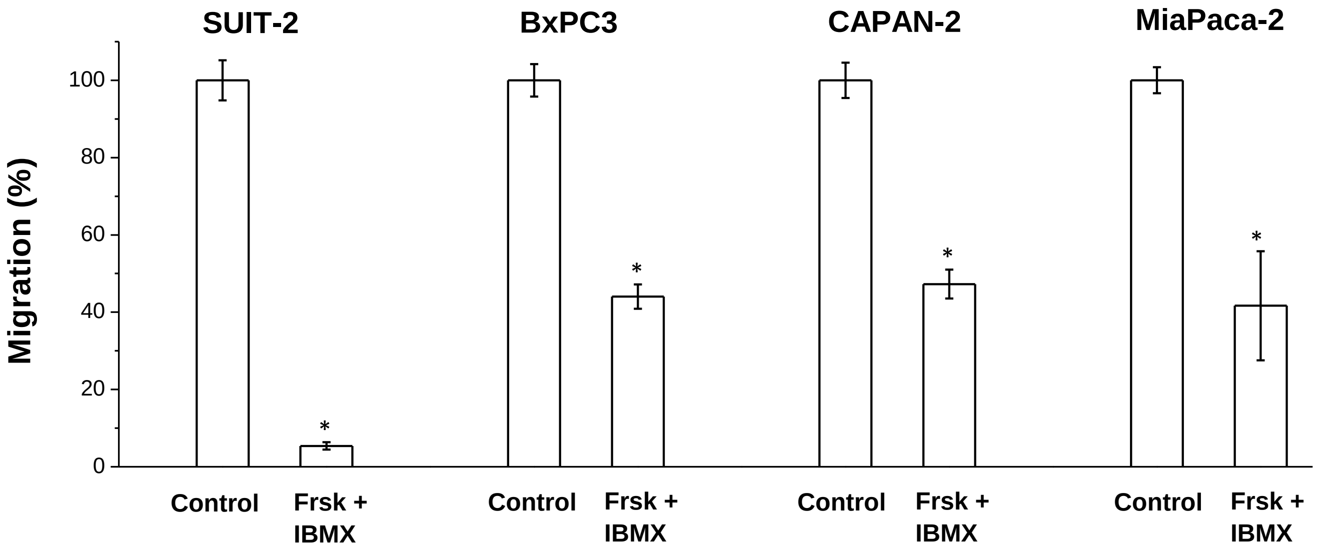

Fig. S2. Forskolin and IBMX inhibit migration of PDAC cells.
Migration of pancreatic ductal adenocarcinoma (PDAC) cell lines SUIT-2 (n=6), BxPC3 (n=9), CAPAN-2 (n=12) and MiaPaca-2 (n=7), as measured by Boyden chamber assays, was inhibited by 20µM forskolin (Frsk) and 1mM IBMX. The difference between the control and treated groups was statistically significant for all cell types.

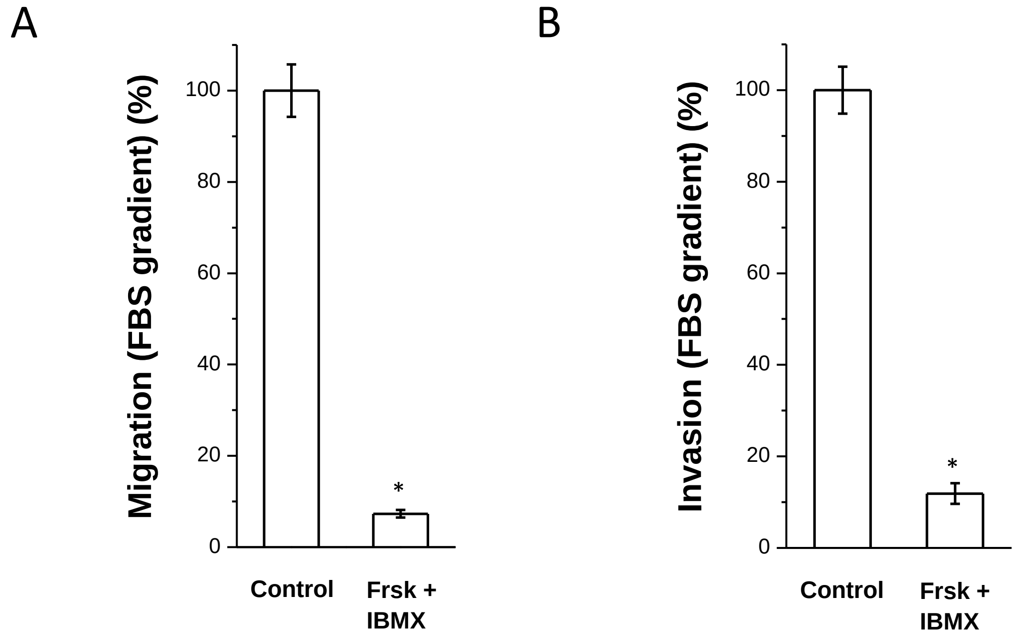

Fig. S3. Forskolin and IBMX inhibit migration and invasion of PANC-1 cells in conditions of asymmetrical FBS.

Cell migration and invasion were assessed in conditions of asymmetric FBS distribution; in these experiments bottom wells were filled with DMEM supplemented with 10% FBS, the top with DMEM alone (i.e. no FBS).

(A) Migration of PANC-1 cells in conditions of asymmetrical FBS, as measured by Boyden chamber assays, was significantly inhibited by 20µM forskolin (Frsk) and 1mM IBMX (n=12).

(B) Invasion of PANC-1 cells in conditions of asymmetrical FBS, as measured by Boyden chamber assays (in these experiments the Boyden chamber membrane was coated with Matrigel), was inhibited by 20µM Frsk and 1mM IBMX (n=6).

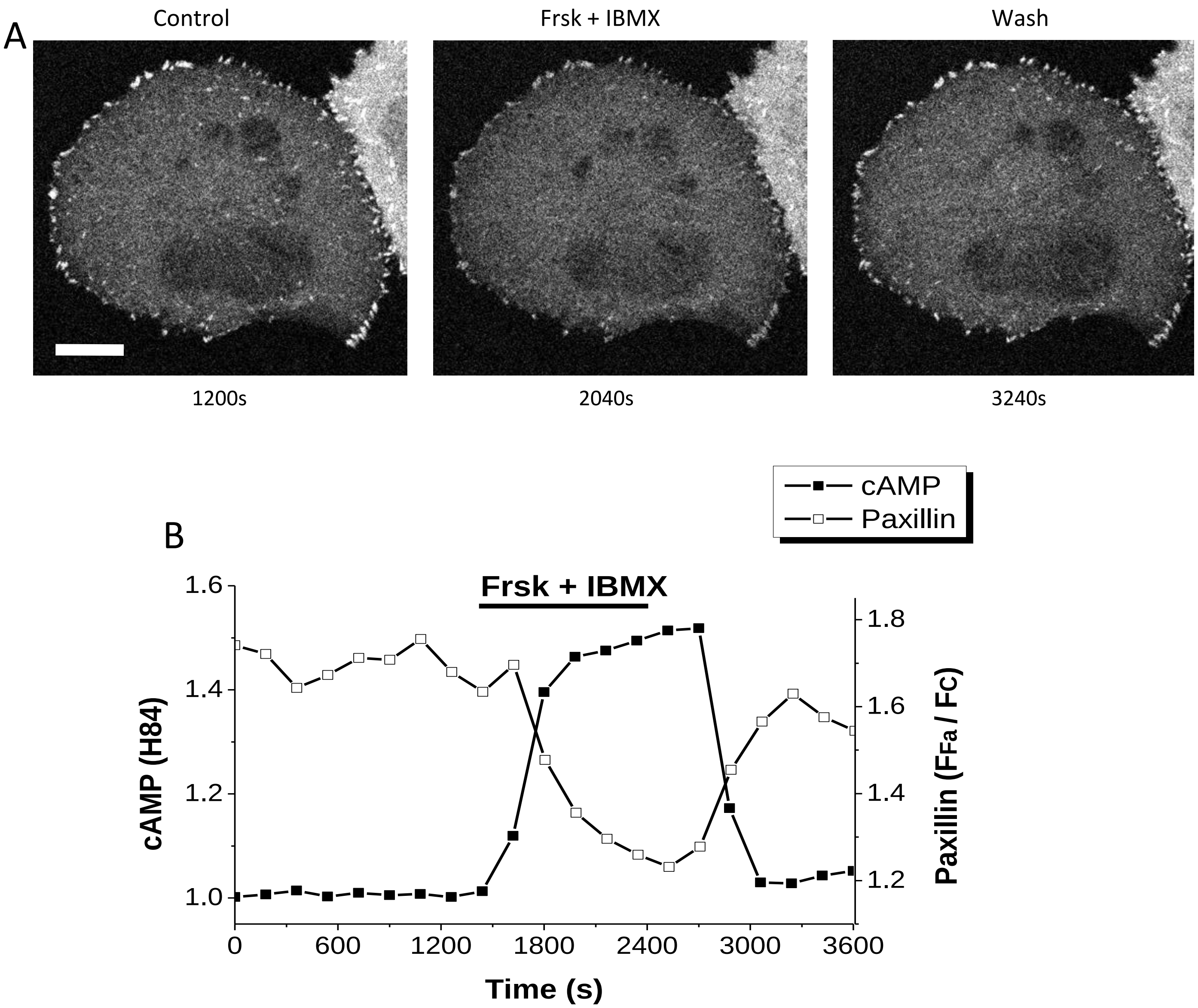

Fig. S4. Correlation between cAMP levels and paxillin trafficking in PANC-1 cells.
(A) Images of the cell transfected with Paxillin-PSmOrange. Note the reversible decrease of fluorescence in focal adhesions upon addition of 20µM forskolin (Frsk) and 1mM IBMX. Scale bar corresponds to 10µm.
(B) The averaged fluorescence recorded from 15 regions containing focal adhesions (F_{Fa}) was divided by fluorescence recorded from the region of the cell devoid of focal adhesions (F_C) and plotted against time (open squares). The cAMP levels were measured from the entire cell and displayed against the time on the same graph (filled squares).
Paxillin-PSmOrange was from Addgene (Addgene plasmid 31923, PI Vladislav Verkhusha) [2]. Notably this plasmid encodes a photoswitchable version of mOrange; however in all our experiments, low intensity laser light was used to ensure that no photoswitching occurred and thus the probe was used as an ordinary mOrange tag with its typical excitation and emission spectra. PSmOrange was excited by 543nm laser and emission collected between 560 and 670nm.

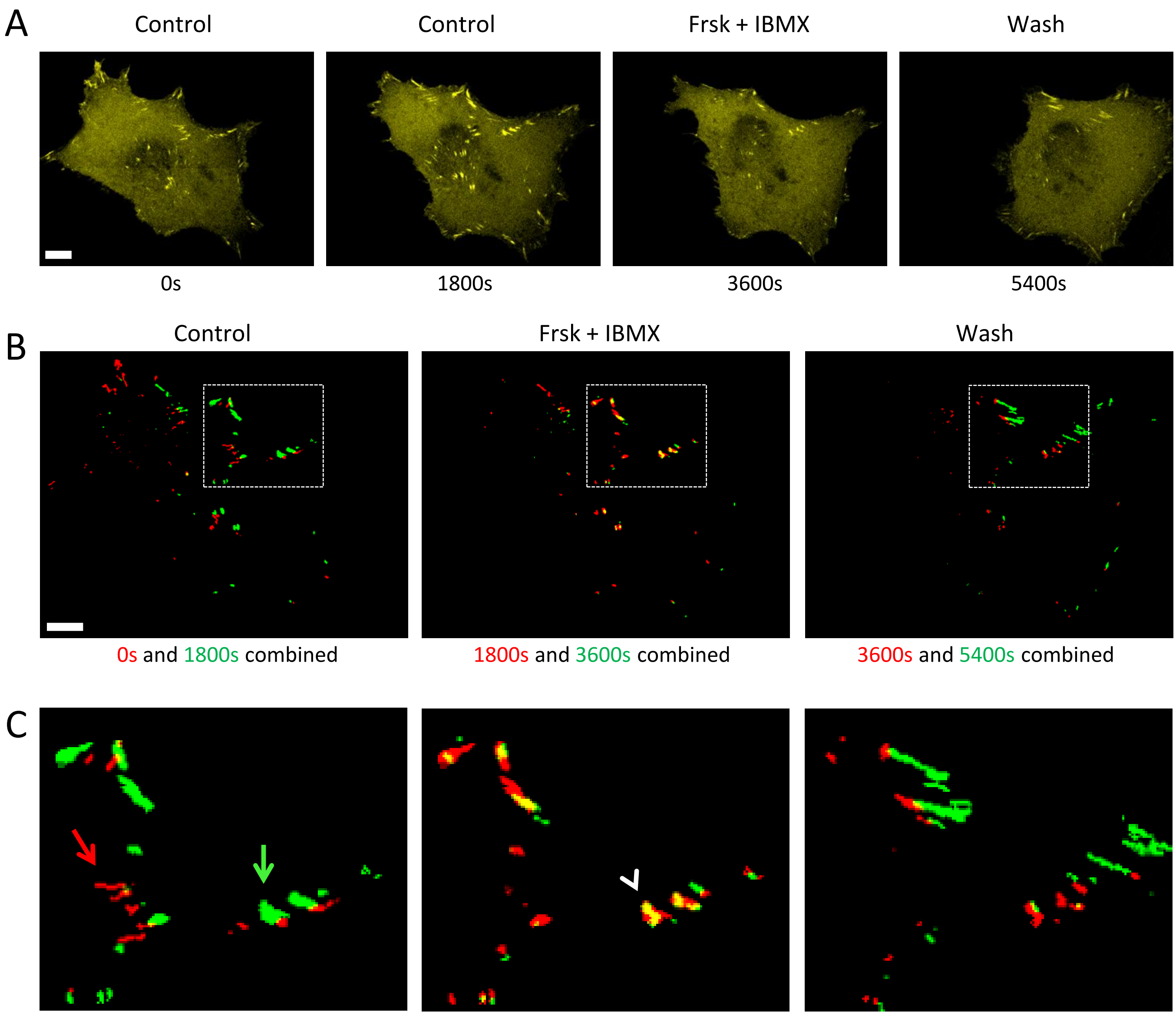

Fig. S5. Increasing cAMP inhibits turnover of focal adhesions revealed by vinculin-venus.
(A) PANC-1 cell transfected with vinculin-venus [3] before (0-1800s), during (1800s-3600s) and after (3600s-5400s, wash period) addition of 20µM Forskolin (Frsk) + 1mM IBMX. The excitation wavelength in these experiments was 514nm and emission was collected between 530nm and 590nm.
(B) The four images from A were thresholded using ImageJ (see Methods) in order to reveal focal adhesions. The two thresholded images from the control period (0s and 1800s, see left panel) were assigned red and green colour respectively and merged together using ImageJ; thus newly formed focal adhesions appear green, while focal adhesions which have disassembled within the 1800s period appear red. Some focal adhesions (or parts of focal adhesions) did not change within the 1800s period and appear yellow as a result of the overlap between the red and green colours. Same process was used to merge thresholded images from 1800s and 3600s during the period of Frsk + IBMX application (see central panel). 1800s image was assigned red colour this time, while 3600s image was coded green. The same process was used again to merge images recorded at 3600s and 5400s (i.e. taken during the wash period; see right panel). Scale bar represents 10µm.
(C) Shows expanded fragment from B (outlined by a box on B). On the left panel green arrow shows an example of newly formed vinculin-venus- labelled focal adhesion; red arrow shows an example of focal adhesion that disappeared during the first 1800s of experiment. Formation of new focal adhesions stopped during Frsk + IBMX treatment. An example of a focal adhesion that was reduced in size but still present at the end of the treatment is shown by white arrowhead. Finally, removal of Forskolin + IBMX restored the formation of new focal adhesions (green - coloured structures on the right panel).

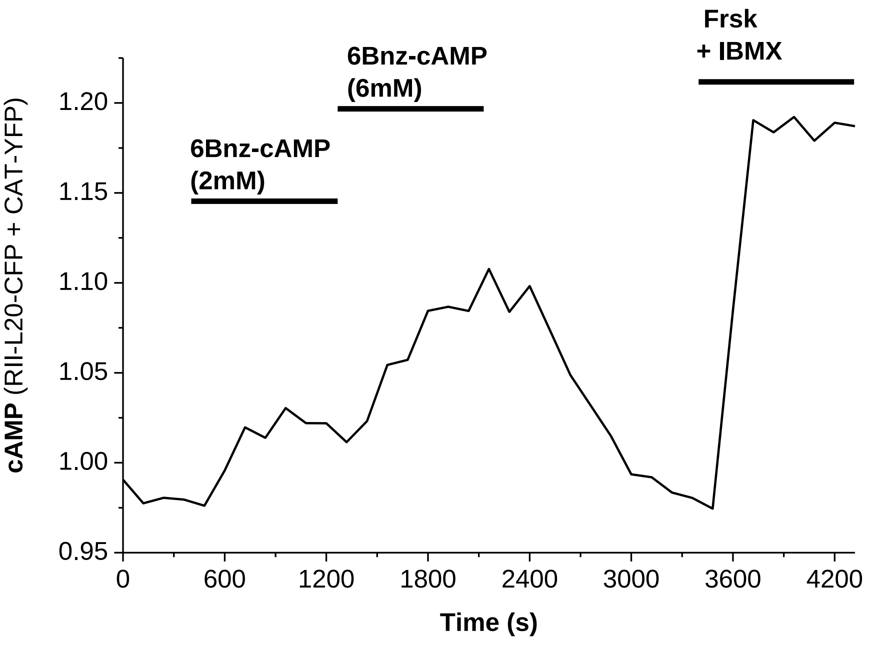

Figure S6. Response of PKA-based sensor to a specific PKA-activating cAMP analogue.
The trace shows the responses of PKA-based cAMP FRET sensor (RII-L20-CFP + CAT-YFP) to the application of 2mM and 6mM N6-benzoyl-cAMP (6Bnz-cAMP, n=18). 20µM forskolin (Frsk) and 1mM IBMX were added at the end of experiments to saturate the probe.
The cAMP FRET sensor [4] was a gift from Dr. M. Zaccolo (University of Oxford).

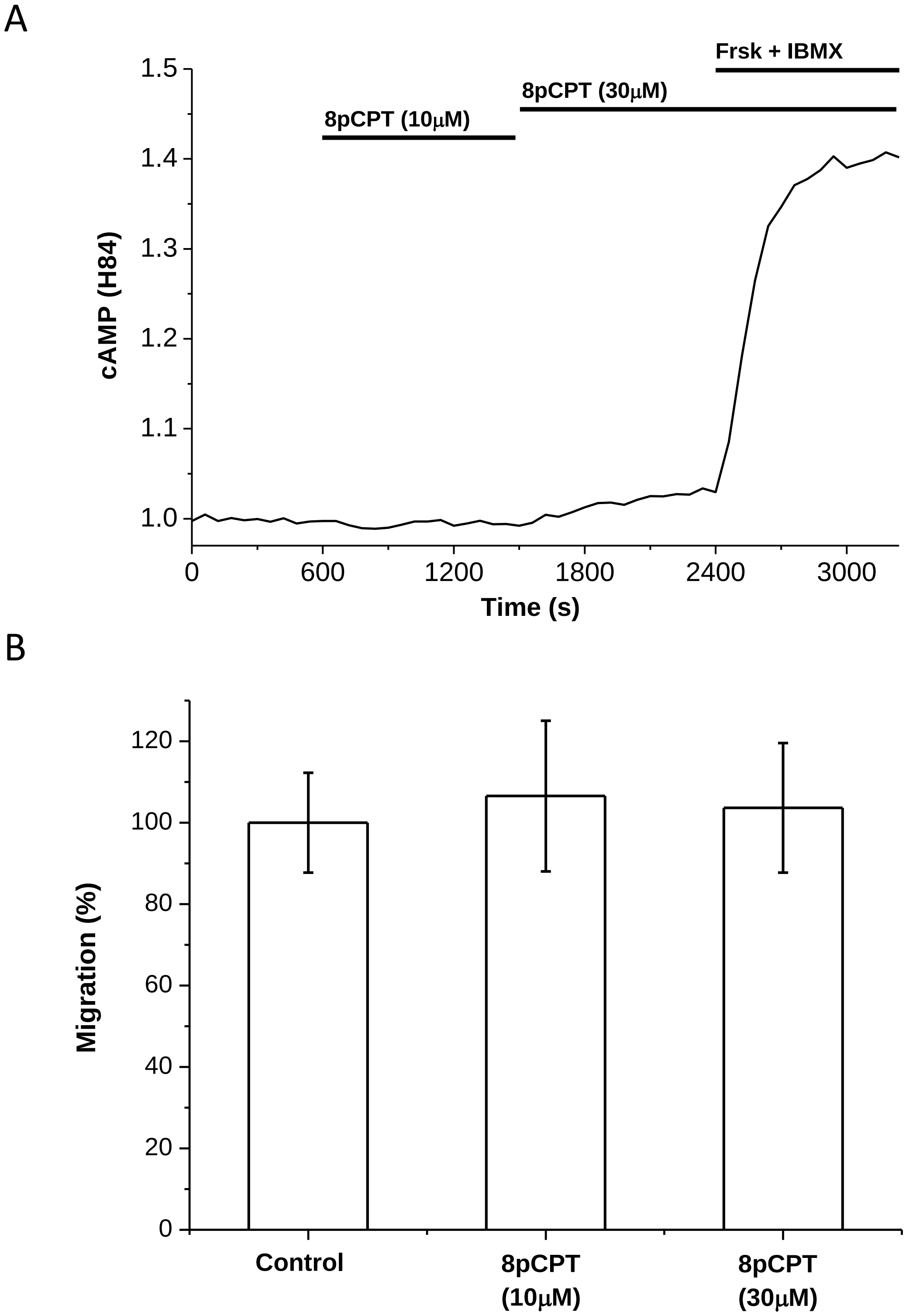

Figure S7. Responses of PANC-1 cells to low doses of a specific EPAC-activating cAMP analogue 8pCPT.

(A) The trace shows a representative response of EPAC-based cAMP FRET sensor (H84) to the application of 10µM and 30µM 8-(4-chlorophenylthio)-2′-O-methyl-cAMP (8pCPT, n=24). 20µM forskolin (Frsk) and 1mM IBMX were added at the end of experiments to saturate the probe. In these experiments 10µM 8pCPT did not produce resolvable responses of H84, however in a majority of cells (n=13 out of n=24) 30 µM 8pCPT produced a small increase in H84 fluorescence ratio.

(B) Migration of PANC-1 cells, as measured by Boyden chamber assays, was not significantly affected by 10µM or 30µM 8pCPT (n=6 for each condition).

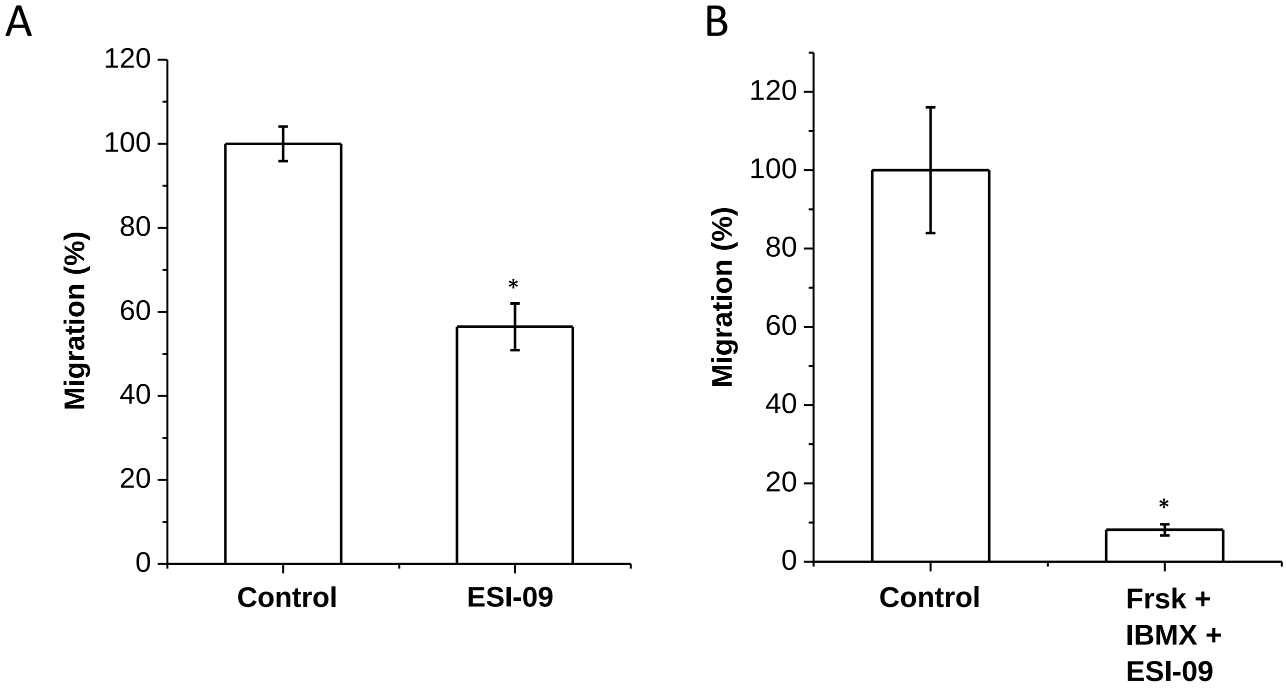

Fig. S8. EPAC-specific inhibitor suppresses migration of PANC-1 cells.
(A) Migration of PANC-1 cells, as measured by Boyden chamber assays, was reduced in the presence of 10µM of EPAC specific inhibitor ESI-09 (n=6 for each condition).
(B) Migration of PANC-1 cells measured in the presence of 10µM ESI-09, was strongly inhibited by a combination of 20µM forskolin (Frsk) and 1mM IBMX (n=6 for each condition). Note that the presence of ESI-09 did not prevent the inhibitory effect of Frsk and IBMX.

Movie S1. Treatment with forskolin and IBMX inhibits ruffle formation and actin dynamics in PANC-1 cells.

Effect of 20µM forskolin (Frsk) and 1mM IBMX on ruffling and actin dynamics of a PANC-1 cell. This movie accompanies Fig.2A of the main part of the manuscript. Top left part (1) shows transmitted light movie of ruffle formation in the cell before, during and after treatment with Frsk and IBMX. Scale bar represents 10µm. The kymograph illustrating the ruffling (shown in the lower left part of the same section) was recorded along the line drawn across the plasma membrane region depicted in the movie. The right part (2) shows the movie (recorded in fluorescence light) illustrating the dynamics of LifeAct-GFP expressed in the same cell. Lower part of this section shows the fluorescence of LifeAct-GFP measured in the same cell along the line drawn across the plasma membrane region (depicted in the movie) and plotted against time. Note the disappearance of ruffles (1) and cessation of actin dynamics (2) following treatment with forskolin and IBMX. Removal of forskolin and IBMX from the extracellular solution restored the ruffling and actin dynamics.

Movie S2. Forskolin and IBMX induce paxillin trafficking from focal adhesions.

Effect of 20µM forskolin (Frsk) and 1mM IBMX on the distribution of Paxillin-GFP fluorescence. This movie accompanies Fig.2C of the main part of the manuscript. Note the decrease of fluorescence in focal adhesions following the application of Frsk and IBMX and increase of the fluorescence after washing off of these compounds. Scale bar corresponds to 10µm.

REFERENCES

1. G.N. van der Krogt, J. Ogink, B. Ponsioen, K. Jalink, A comparison of donor-acceptor pairs for genetically encoded FRET sensors: application to the Epac cAMP sensor as an example, PLoS. One. 3 (2008) p.e1916.

2. O.M. Subach, D. Entenberg, J.S. Condeelis, V.V. Verkhusha, A FRET-facilitated photoswitching using an orange fluorescent protein with the fast photoconversion kinetics, J. Am. Chem. Soc. 134 (2012) pp. 14789-14799.

3. C. Grashoff, B.D. Hoffman, M.D. Brenner, R. Zhou, M. Parsons, M.T. Yang, M.A. McLean, S.G. Sligar, C.S. Chen, T. Ha, M.A. Schwartz, Measuring mechanical tension across vinculin reveals regulation of focal adhesion dynamics, Nature 466 (2010) pp. 263-266.

4. V. Lissandron, A. Terrin, M. Collini, L. D'alfonso, G. Chirico, S. Pantano, M. Zaccolo, Improvement of a FRET-based indicator for cAMP by linker design and stabilization of donor-acceptor interaction, J. Mol. Biol. 354 (2005) pp. 546-555
