# Supplementary figures and images for: cAMP inhibits migration, ruffling and paxillin accumulation in focal adhesions of pancreatic ductal adenocarcinoma cells: Effects of PKA and EPAC
Source: Biochim Biophys Acta. 2013 Dec;1833(12):2664–72. doi: 10.1016/j.bbamcr.2013.06.011 (PMC3898478; doi:10.1016/j.bbamcr.2013.06.011)

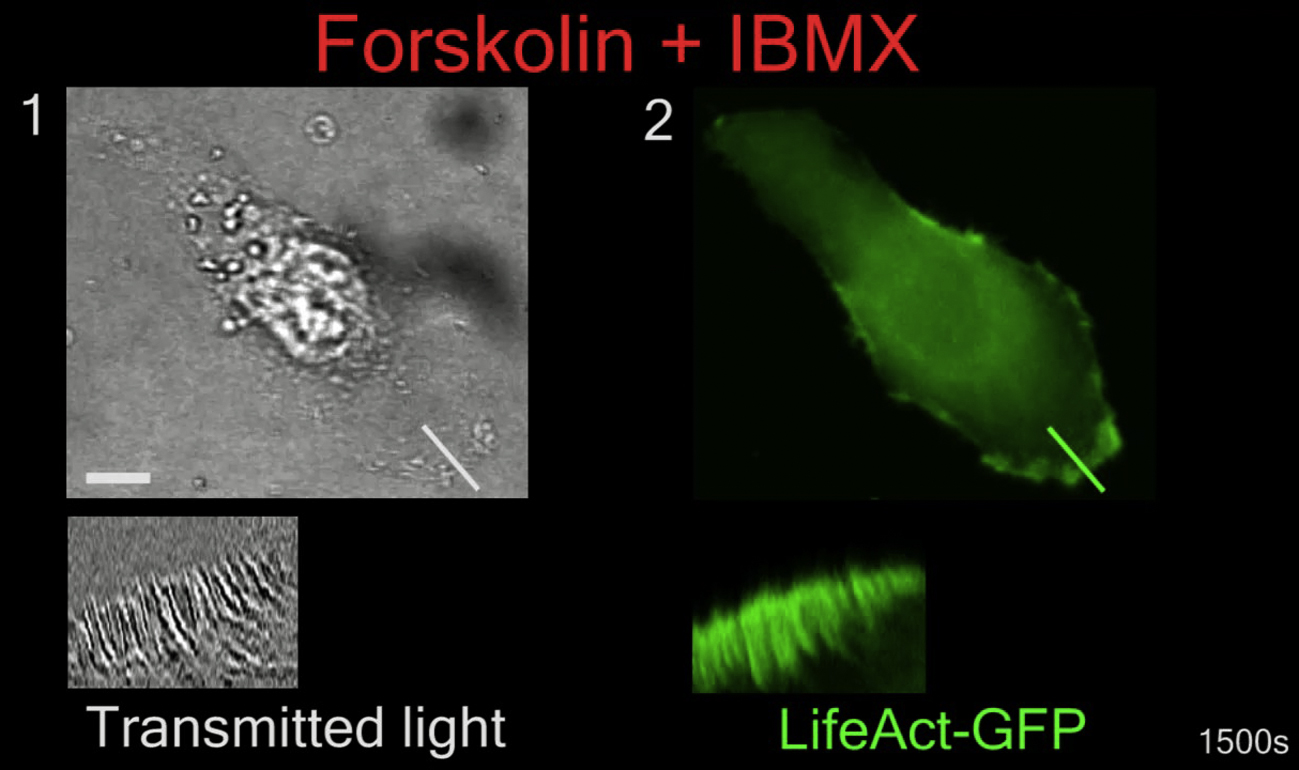

Supplement: Movie S1 — Treatment with forskolin and IBMX inhibits ruffle formation and actin dynamics in PANC-1 cells. Effect of 20 μM forskolin (Frsk) and 1 mM IBMX on ruffling and actin dynamics of a PANC-1 cell. This movie accompanies Fig. 2A of the main part of the manuscript. Top left part (1) shows transmitted light movie of ruffle formation in the cell before, during and after treatment with Frsk and IBMX. Scale bar represents 10 μm. The kymograph illustrating the ruffling (shown in the lower left part of the same section) was recorded along the line drawn across the plasma membrane region depicted in the movie. The right part (2) shows the movie (recorded in fluorescence light) illustrating the dynamics of LifeAct-GFP expressed in the same cell. Lower part of this section shows the fluorescence of LifeAct-GFP measured in the same cell along the line drawn across the plasma membrane region (depicted in the movie) and plotted against time. Note the disappearance of ruffles (1) and cessation of actin dynamics (2) following treatment with forskolin and IBMX. Removal of forskolin and IBMX from the extracellular solution restored the ruffling and actin dynamics. [file mmc2.jpg]

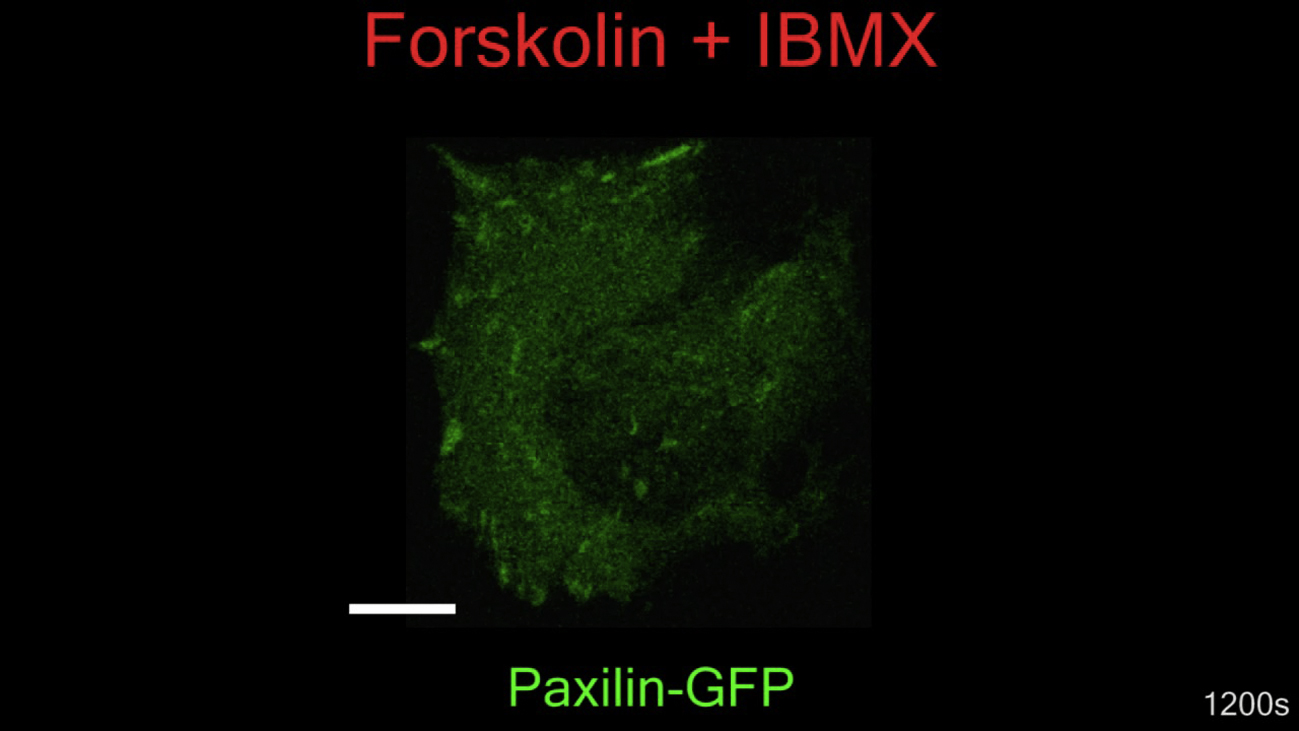

Supplement: Movie S2 — Forskolin and IBMX induce paxillin trafficking from focal adhesions. Effect of 20 μM forskolin (Frsk) and 1 mM IBMX on the distribution of paxillin–GFP fluorescence. This movie accompanies Fig. 2C of the main part of the manuscript. Note the decrease of fluorescence in focal adhesions following the application of Frsk and IBMX and increase of the fluorescence after washing off of these compounds. Scale bar corresponds to 10 μm. [file mmc3.jpg]
